# Integrated study of systemic and local airway transcriptomes in asthma reveals causal mediation of systemic effects by airway key drivers

**DOI:** 10.1186/s13073-023-01222-2

**Published:** 2023-09-20

**Authors:** Lingdi Zhang, Yoojin Chun, Haritz Irizar, Zoe Arditi, Galina Grishina, Alexander Grishin, Alfin Vicencio, Supinda Bunyavanich

**Affiliations:** 1 Department of Genetics and Genomic Sciences, Icahn School of Medicine at Mount Sinai, 1425 Madison Avenue, New York, NY 10029 USA; 2 Division of Allergy and Immunology, Department of Pediatrics, Icahn School of Medicine at Mount Sinai, 1425 Madison Avenue, New York, NY 10029 USA

**Keywords:** Asthma, Transcriptome, Blood, Airway, Nasal, Peripheral blood mononuclear cell, NK cell, Interleukin, Tricarboxylic acid, Causal network

## Abstract

**Background:**

Systemic and local profiles have each been associated with asthma, but parsing causal relationships between system-wide and airway-specific processes can be challenging. We sought to investigate systemic and airway processes in asthma and their causal relationships.

**Methods:**

Three hundred forty-one participants with persistent asthma and non-asthmatic controls were recruited and underwent peripheral blood mononuclear cell (PBMC) collection and nasal brushing. Transcriptome-wide RNA sequencing of the PBMC and nasal samples and a series of analyses were then performed using a discovery and independent test set approach at each step to ensure rigor. Analytic steps included differential expression analyses, coexpression and probabilistic causal (Bayesian) network constructions, key driver analyses, and causal mediation models.

**Results:**

Among the 341 participants, the median age was 13 years (IQR = 10–16), 164 (48%) were female, and 200 (58.7%) had persistent asthma with mean Asthma Control Test (ACT) score 16.6 (SD = 4.2). PBMC genes associated with asthma were enriched in co-expression modules for NK cell-mediated cytotoxicity (fold enrichment = 4.5, FDR = 6.47 × 10^−32^) and interleukin production (fold enrichment = 2.0, FDR = 1.01 × 10^−15^). Probabilistic causal network and key driver analyses identified NK cell granule protein (*NKG7*, fold change = 22.7, FDR = 1.02 × 10^−31^) and perforin (*PRF1*, fold change = 14.9, FDR = 1.31 × 10^−22^) as key drivers predicted to causally regulate PBMC asthma modules. Nasal genes associated with asthma were enriched in the tricarboxylic acid (TCA) cycle module (fold enrichment = 7.5 FDR = 5.09 × 10^−107^), with network analyses identifying G3BP stress granule assembly factor 1 (*G3BP1*, fold change = 9.1 FDR = 2.77 × 10^−5^) and InaD-like protein (*INADL*, fold change = 5.3 FDR = 2.98 × 10^−9^) as nasal key drivers. Causal mediation analyses revealed that associations between PBMC key drivers and asthma are causally mediated by nasal key drivers (FDR = 0.0076 to 0.015).

**Conclusions:**

Integrated study of the systemic and airway transcriptomes in a well-phenotyped asthma cohort identified causal key drivers of asthma among PBMC and nasal transcripts. Associations between PBMC key drivers and asthma are causally mediated by nasal key drivers.

**Supplementary Information:**

The online version contains supplementary material available at 10.1186/s13073-023-01222-2.

## Background

Asthma is a chronic respiratory disease that affects millions of people of all ages worldwide [1, 2]. Individuals with asthma experience wheezing, cough, chest tightness, and/or shortness of breath that can lead to impaired quality of life, emergency department visits, hospitalizations, and mortality [3]. Although important progress has been made in asthma research, many gaps remain in our mechanistic understanding of this common disease [4].

Asthma is a heterogeneous disorder of airway hyperresponsiveness [3] where the presence of inflammatory cells including type 2 cells, eosinophils, and basophils in the local airway is common [5]. Transcriptome studies of upper airway samples from individuals with asthma have helped to characterize local biology associated with asthma and asthma-related phenotypes [6–12]. However, measures of systemic inflammation, such as eosinophilia and neutrophilia, have also been associated with asthma and asthma phenotypes [13–15]. Local inflammation can be sensed by hematopoietic progenitor cells in the bone marrow, which leads to increased programming of myeloid cells that enter the circulation [16]. The recruitment of circulating immune cells to the airway during inflammation [13] is a cross-talk that bridges systemic and local inflammation. These prior studies suggest a role for systemic inflammatory processes in asthma, which have been further examined via blood transcriptome-based investigations [17–19]. Interestingly, although airway and blood gene expression associations with asthma have been respectively reported, prior investigations of asthma phenotypes that concurrently examined blood and airway samples found differential gene expression in the airway only with no detectable differences in blood [20, 21]. The details and causal relationships between systemic and local transcriptomics in asthma merit further examination.

In this study, we hypothesized that (1) both systemic and local gene expression are associated with asthma, and (2) systemic inflammation associated with asthma is causally mediated by airway gene expression. Leveraging parallel peripheral blood mononuclear cell (PBMC) and nasal transcriptome data generated from 341 individuals and using causal network approaches with discovery and tests sets to ensure rigor, we identified, validated, and characterized systemic and local gene signatures of asthma as well as causal relationships between their key drivers.

## Methods

**Fig. 1:**
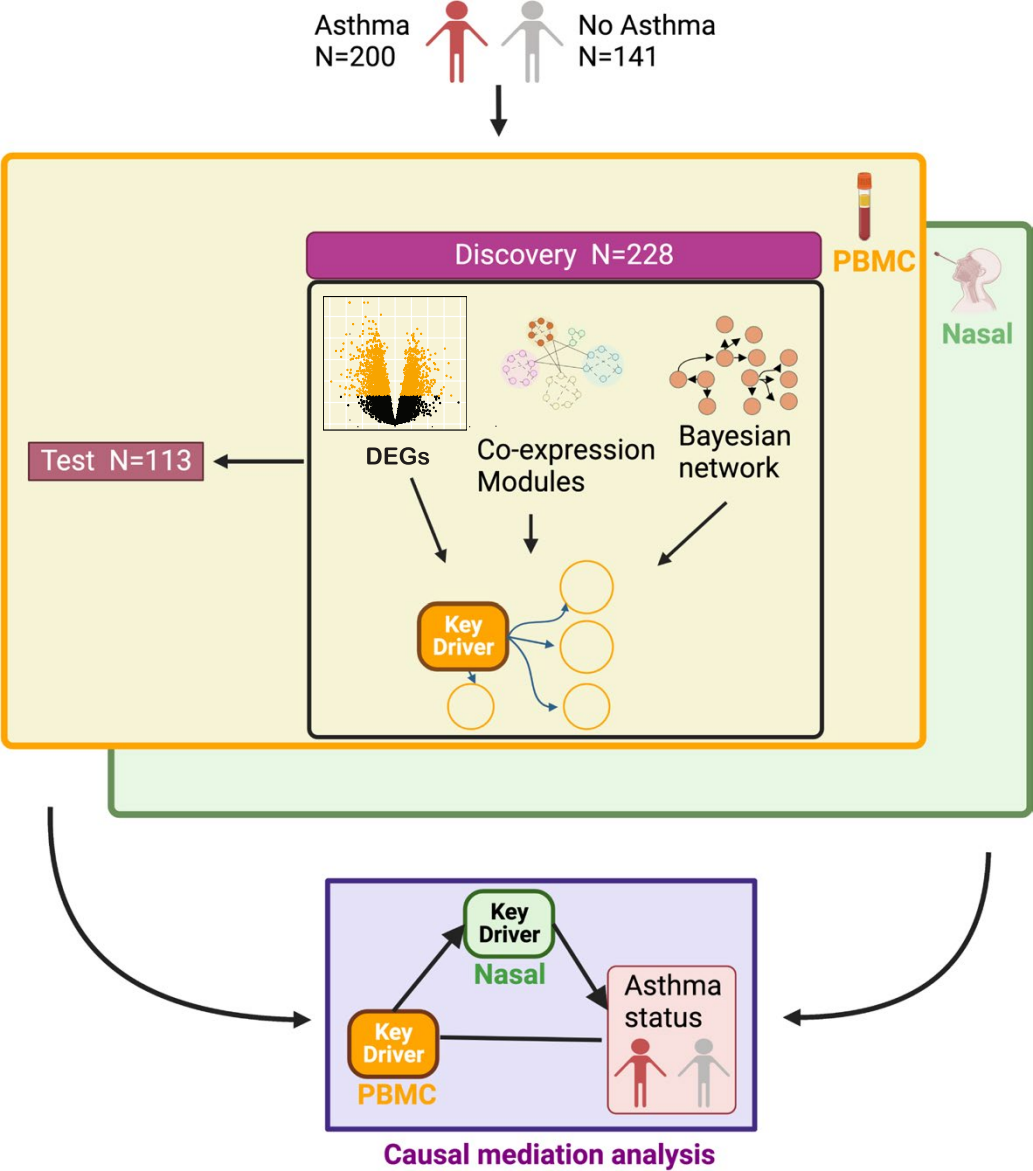
Study flow. Peripheral blood mononuclear cell (PBMC) and nasal transcriptome profiles from 341 participants with and without asthma were generated and studied to characterize systemic and airway responses in asthma. Using PBMC transcriptome data from the discovery set, we first identified differentially expressed genes (DEGs) associated with asthma (yellow box). Genes that were also associated with asthma with the same direct of effect in the independent test set were deemed validated “PBMC asthma genes.” Weighted gene coexpression network analysis and enrichment testing were then performed to identify co-expression modules enriched for PBMC asthma genes (PBMC asthma modules). Next, probabilistic causal (Bayesian) networks were built separately for the discovery set and test set. Key driver analysis was performed on each network using PBMC asthma module members as targets. Key drivers identified in both the discovery and test sets were deemed “PBMC key drivers.” The same series of analyses was performed with the nasal transcriptome data generated in parallel from participants to identify nasal asthma genes, nasal asthma modules, and nasal key drivers (green box). To characterize relationships between the PBMC key drivers and nasal key drivers identified, causal mediation analyses were then performed (purple box)

Figure 1 provides an overview of the study flow. The study encompassed recruitment and sample collection from 341 participants followed by transcriptome data generation, discovery and test set assignment, differential gene expression and weighted gene coexpression network analyses, cellular deconvolution, and probabilistic causal (Bayesian) network and key driver analyses. These steps were carried out for PBMC and nasal samples in parallel, and key drivers identified from the PBMC and nasal data were then jointly analyzed in causal mediation models (Fig. 1). The methods for each of these steps are detailed below.

### Study population and sample collection

Three hundred forty-one participants with and without asthma were recruited from the Mount Sinai Health System, New York, NY. All participants or parents of minors provided written informed consent for study participation. The study conformed to the principles of the Helsinki Declaration and was approved by the Mount Sinai Institutional Review Board (Study 15–00202). Inclusion criteria included those with persistent asthma (based on physician diagnosis, asthma symptoms ≥ 2x/week, and demonstration of a bronchodilator response or positive methacholine challenge) and controls without asthma. Non-asthmatic controls had normal spirometry with no bronchodilator response and no personal nor family history of asthma. Questionnaires addressing asthma-related symptoms and history were completed by all participants. Allergen sensitization by serum specific IgE measurement to 10 environmental allergens, the asthma control test (ACT) [22], pre- and post-bronchodilator spirometry lung function testing following American Thoracic Society guidlines [23], and peripheral blood collection were also performed on all subjects. All participants were off asthma medications for at least 4 weeks and nasal medications for at least 2 weeks at the time of sampling. PBMCs were immediately isolated from whole blood samples by Ficoll-Paque density gradient centrifugation and then cryopreserved. Additionally, all subjects were invited to undergo nasal brushing with a sterile cytology brush, with 292 subjects (Additional file 1: Table S1) agreeing to this additional procedure. Nasal brushings were immediately placed in RNALater (Qiagen, Valencia, CA) and stored at – 80 °C.

### Transcriptome data generation

RNA from PBMC and nasal samples was extracted using Qiagen RNeasy Mini Kit (Valencia, CA). RNA quality and quantity were measured using 2100 Bioanalyzer and Qubit fluorometer. Sequencing libraries for PBMC RNA were prepared using the Ribo-Zero Gold kit (Illumina) and sequenced on Illumina NovaSeq (Illumina) with paired-end 150 bp reads generated with 40–50 million reads per sample. Sequencing libraries for nasal RNA were prepared using TruSeq RNA Sample Prep Kit v2 protocol (Illumina) and sequenced on Illumina HiSeq 2500. Paired-end 100 bp reads were generated with 40–50 million reads per sample. The reads were mapped to GRCH38 using STAR v2.4.0g1 aligner, and the number of reads mapped per gene was estimated using HTseq [24]. The gene expression profiles were normalized to counts per million (cpm) using edgeR r package [25]. Genes with ≤ 5 counts per million in > 10% of samples were removed to reduce noise on low counts and low abundance genes. After quality control and filtering, 12484 PBMC genes and 13996 nasal genes remained for analysis.

### Discovery and test set assignment

The 341 participants were randomly split 2:1 into a discovery set (*n* = 228) and test set (*n* = 113). At each major step, the discovery set was used for initial models, and significant findings (FDR ≤ 0.05) that could also be found in the test set of independent participants were considered validated and carried forward.

### Differential gene expression analysis and WGCNA

DESeq2 [26] was used to identify PBMC and nasal genes associated with asthma. Age, sex, and race were included as covariates in the models. Genes with Benjamini–Hochberg corrected *p* values ≤ 0.05 in the discovery set and associated with asthma with the same direction of effect in the test set were deemed validated PBMC or nasal asthma genes, respectively.

Weighted gene coexpression network analysis (WGCNA) [27] was used to build PBMC and nasal gene co-expression networks separately. PBMC and nasal gene co-expression modules enriched for PBMC or nasal asthma genes were then identified by Fisher’s exact test (enrichment score > 1 and FDR ≤ 0.05). Gene ontology (GO) analysis was performed on each module’s gene set using DAVID [28], and biological processes ranked by fold enrichment and FDR ≤ 0.05 were selected as module names.

### Cellular deconvolution

Cellular composition of the nasal samples was inferred by deconvolution where CIBERSORTx [29] analysis was performed on the nasal RNAseq data with nasal single cell RNAseq data [30] as the reference.

### Probabilistic causal network and key driver analysis

Probabilistic causal (Bayesian) networks [31, 32] for the PBMC and nasal transcriptome data were built separately for each’s respective discovery and test sets using RIMBAnet [31, 32], a software to construct probabilistic causal networks using Markov chain Monte Carlo simulations. Inputs included the corresponding PBMC or nasal transcriptome data and eQTL data [33]. To map eQTLs for the PBMC and nasal transcriptomes respectively, two-path mode from STAR aligner [34] was used to align RNAseq reads to the reference genome (GRCh38), and variants were called using the “HaplotypeCaller” GATK [35] tool after filtering against known RNA editing sites, intronic variants within four nucleotides from splice donor, and acceptor site variants called in repeated sequences. We performed genotype phasing and imputation using BEAGLE 5.1 [36] against the 1000 Genome reference haplotypes for hg38 [37]. After filtering out variants with low imputation accuracy (dosage R-squared < 0.8) and low minor allele frequency (MAF < 0.01), we hard called the variants using PLINK2 [38]. Genetic ancestry of the study population was calculated based on the variant calls [39], and this genetic ancestry together with age and gender were used as covariates when mapping cis eQTLs [33, 40]. We identified the eQTLs where cis SNPs (within 1 Mb of the transcription start or end site of the gene) were associated with gene expression at FDR ≤ 0.05 using matrixeqtl [40] (Additional file 1: Tables S2-S4).

To construct the probabilistic causal networks, genes from the corresponding transcriptomic datasets were discretized into 3 states, high expression, low expression, and no expression by K-means clustering (*K* = 3) [31, 32, 41]. Markov chain Monte Carlo simulations were used to reconstruct 1000 networks, and the fit of each network was assessed by the Bayesian Information Criterion [31, 32, 41]. The final causal network was built by retaining edges present in ≥ 30% of the 1000 networks.

Key driver analysis using the KDA package [42] was then performed to identify each asthma module’s key drivers using the causal network and module members as targets. Subnetworks used as background for the enrichment analysis were identified by selecting nodes K-steps away from the nodes in the module member list. The enrichment of module member genes was assessed in k-step (k varies from 1 to K) downstream neighborhood stemming from each node. We used *K* = 7 in the analyses.

### Causal mediation analysis

PBMC and nasal key drivers associated with asthma were tested in causal mediation analysis using the robust structural modeling equation implemented in the lavaan R package [43]. We tested (a) the degree to which PBMC key drivers (mediator) mediated the effects of nasal key drivers (independent variable) on asthma (outcome) and (b) the degree to which nasal key drivers (mediator) mediated the effects of PBMC key drivers (independent variable) on asthma (outcome). For each causal mediation analysis, three regression models were implemented to estimate the effects of (1) independent variables on outcomes, (2) independent variables on mediators, and (3) mediators on the outcomes. Model 1 estimates the direct effect of independent variables on the outcome, while models (2) and (3) estimate indirect effects (mediation). FDR $\le$ 0.05 was used as the threshold for significance.

## Results

### Participant characteristics

The cohort included 341 participants recruited from the Mount Sinai Health System, New York, USA, of whom 200 (58.7%) individuals had persistent asthma based on physician diagnosis, symptoms ≥ 2x/week, and demonstration of bronchodilator response on lung function testing and/or positive methacholine challenge. One hundred forty-one (41.3%) individuals had no asthma based on no personal or family history of asthma and demonstration of normal spirometry without a bronchodilator response (Table 1). Participants were primarily children with median age of 13 years (IQR 10–16). Those with asthma were younger, more likely to be Black or Latino, and had lower ACT scores, FEV1%, and FEV1/FVC values. Many participants with asthma had high rates of emergency department visits and hospitalizations for asthma (Table 1). Peripheral blood for PBMC isolation was collected from all 341 participants. Nasal brushings were collected from a subset (*n* = 292, 85.6%) who agreed to nasal brushing; the characteristics of this subset did not significantly differ from the whole cohort (Additional file 1: Table S1).

**Table 1:** Characteristics of the cohort

	All (*N* = 340)	Asthma *N* = 200	No asthma *N* = 141	*p* value
Age, mean (SD)	13.4 (4.8)	12.5 (4.6)	14.7 (5.0)	4.90E − 05
Sex female, no. (%)	164 (48%)	83 (42%)	81 (57%)	0.0042
Race/ethnicity, no. (%)				3.93E − 06
Asian	23 (6.7%)	4 (2.0%)	19 (13.5%)	
Black	61 (17.9%)	43 (21.5%)	18 (12.8%)	
Latino	86 (25.2%)	63 (31.5%)	23 (16.3%)	
Multiple races or unknown	30 (8.8%)	15 (7.5%)	15 (10.6%)	
White	141 (41.3%)	75 (37.5%)	66 (46.8%)	
^a^Asthma medications prescribed, no. (%)				
β-Agonist	190 (56.0%)	190 (95.0%)	0 (0%)	
ICS	44 (13.0%)	44 (22.0%)	0 (0%)	
ICS/long-acting β-antagonist	55 (16.1%)	55 (27.5%)	0 (0%)	
Leukotriene receptor antagonist	47 (13.8%)	47 (23.5%)	0 (0%)	
Omalizumab	5 (1.5%)	5 (2.5%)	0 (0%)	
ACT score, mean (SD)	20.1 (5.3)	16.6 (4.2)	25 (0.0)	< 2e − 16
FEV1% predicted, mean (SD)	88 (15.4)	86 (17.0)	90 (13.1)	0.022
FEV1/FVC ratio, mean (SD)	83.5 (10.9)	79.7 (10.3)	87.6 (10.0)	1.36E − 10
Allergen sensitization, no. (%)	261 (76.5%)	146 (73%)	115 (82%)	0.067
Hospitalizations for asthma in the past year, no. (%)				
0	174 (51%)	174 (87%)	NA	
1	11 (3.2%)	11 (5.5%)	NA	
≥ 2	15 (4.4%)	15 (7.5%)	NA	
Emergency dept visits for asthma in the past year, no. (%)				
0	132 (38.7%)	132 (66%)	NA	
1	20 (5.9%)	20 (10%)	NA	
≥ 2	48 (14.1%)	48 (24%)	NA	
Rhinosinusitis symptoms, no. (%)	111 (32.6%)	82 (41.0%)	29 (20.6%)	9.89e − 5

*P* values were calculated using Fisher’s exact test for categorical variables and generalized linear regression for continuous variables

*ACT* asthma control test, *FEV1% *force expiratory volume in 1 s, percent predicted, *FEV1/FVC* forced expiratory volume in 1 s/forced vital capacity

^a^Asthma medications were held for at least 4 weeks before sampling

### Systemic processes in asthma: PBMC asthma genes are enriched in co-expression modules for NK cell-mediated cytotoxicity and interleukin production

**Fig. 2:**
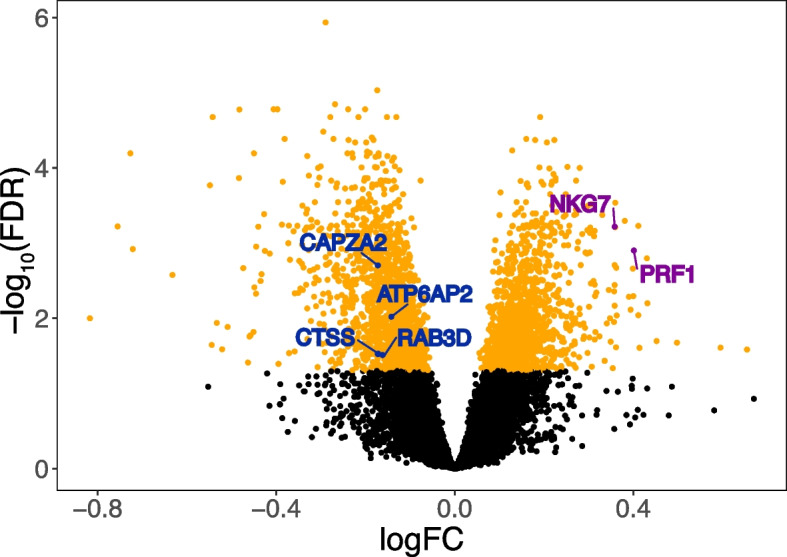
PBMC genes associated with asthma. Differential gene expression analysis was performed. Genes associated with asthma in the discovery set (FDR $\le$ 0.05) are shown in orange, and those that were also associated with asthma with the same direction of effect in the independent test set were deemed validated PBMC asthma genes. Key drivers identified and validated in downstream analyses are labeled, including key drivers of the NK cell-mediated cytotoxicity module (purple) and key drivers of the interleukin production module (blue)

To investigate systemic processes in asthma, we isolated PBMCs from participants’ peripheral blood and generated PBMC transcriptome profiles using RNA sequencing [44]. The 341 participants were randomly split into a discovery set (*n* = 228) and test set (*n* = 113). To identify PBMC transcripts significantly associated (FDR ≤ 0.05) with asthma, differential gene expression analysis on the discovery set was performed with age, sex, and race/ethnicity as covariates. PBMC transcripts associated with asthma in the discovery set (Fig. 2) were then tested in the independent test set, and those that were also associated with the same direction of effect in the test set were deemed validated “PBMC asthma genes” (Additional file 1: Table S5).

We then sought to uncover functional biological context for the identified PBMC asthma genes. Weighted gene co-expression network analysis (WGCNA) [27] using the discovery set identified 24 PBMC co-expression modules representing broader constructs of biological function. Nine of these 24 modules were significantly enriched (Fisher’s exact test FDR ≤ 0.05) for PBMC asthma genes. Gene Ontology (GO) enrichment analyses revealed that the modules most strongly enriched for PBMC asthma genes (ranked by effect size and then lowest FDR) were the modules for “NK cell-mediated cytotoxicity” (fold enrichment = 4.5, FDR = 6.47 × 10^−32^) and “interleukin production” (fold enrichment = 2.0, FDR = 1.01 × 10^−15^). We will henceforth refer to these two modules as the NK cell and interleukin PBMC asthma modules, and their respective member genes are shown in Additional file 1: Tables S6, S7). The remaining seven modules largely represented cellular maintenance processes. Notably, genes in causal paths for Th2 cell differentiation regulation gene ontology process were enriched in the interleukin PBMC asthma module (enrichment score 19.0, Fisher’s exact *p* value = 7.24e − 23).

### Causal relationships and key drivers of the PBMC asthma modules

To characterize causal relationships among genes within the identified PBMC asthma modules, we next performed probabilistic causal (Bayesian) network analysis [31, 32, 45]. In this network construction, causal relationships were statistically inferred between PBMC transcripts using expression quantitative trait loci mapped in this cohort as priors [31–33] (Additional file 1: Tables S3-S4). We then used key driver analysis (KDA) [42], a method for applying dynamic and statistical neighborhood searches on constructed probabilistic causal networks, to identify key drivers predicted to exert the greatest causal impact on downstream genes in each PBMC asthma module. Here, we also used a discovery and test set approach, where a probabilistic causal network was first built with the discovery set and key drivers were identified for each PBMC asthma module (Additional file 1: Table S8, Table S9). Independent probabilistic causal network construction with the test set and key driver analysis were then separately performed for each module. Key drivers identified in the discovery set and also found in the test set were deemed validated “PBMC key drivers” of each module.

**Fig. 3:**
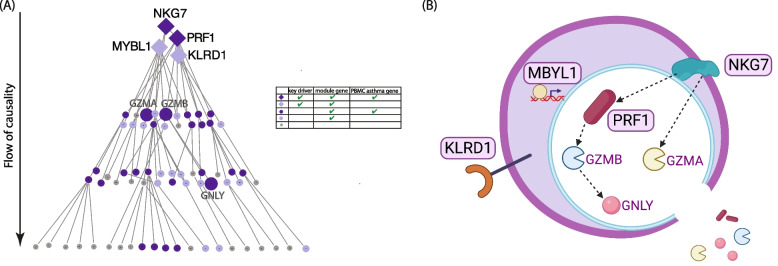
Probabilistic causal network and functional biological context for the NK cell-mediated cytotoxicity PBMC module. **A** Probabilistic causal network and key driver analysis results for the NK cell-mediated cytotoxicity module. This PBMC transcriptome module was significantly enriched with PBMC asthma genes. The arrow indicates the overall causality flow with key drivers at the top level. Level indicates path length of the gene from a key driver. Genes on higher levels have greater causal impact on downstream genes. Color, shade, and shape indicate key driver, module membership, and PBMC asthma genes as summarized in the legend. Selected non-key drivers are additionally labeled, as they are recognized to function with the upstream key drivers. **B** Functional biological context for the NK cell-mediated cytotoxicity module. Genes within purple boxes are key drivers and genes in purple font are module genes highlighted in **A**. Dashed arrows indicate causal relationships inferred from the causal network. *NKG7* encodes natural killer cell granule protein 7, which regulates granule exocytosis in lymphocytes [48]. *PRF1* encodes perforins that function with granzymes and granulysins to kill target cells [47]. Killer cell lectin-like receipt D1 encoded by *KLRD1* forms heterodimers with NKG2 and can stimulate or inhibit cytotoxicity depending on NKG2 isoform [58, 59]. *MBYL1*, MYB proto-oncogene like 1, is a transcription activator [53]. The module genes *GZMB, GZMA* and *GNLY* encode granzymes and granulysin that kill target cells upon release [47, 49]

**Fig. 4:**
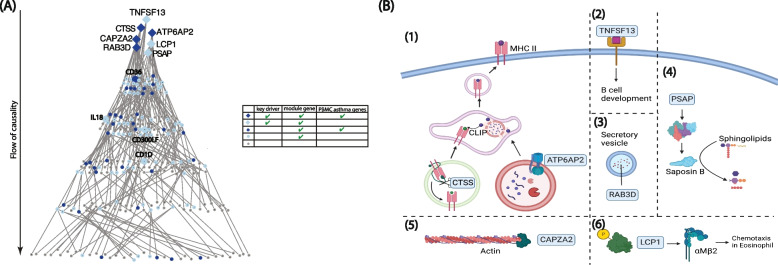
Probabilistic causal network and functional biological context for the interleukin production PBMC module. **A** Probabilistic causal network and key driver analysis results for the interleukin production module. This PBMC transcriptome module was significantly enriched with PBMC asthma genes. The arrow indicates the overall causality flow with the key drivers on the top level. Level indicates path length of the gene from a key driver. Genes on higher levels have greater causal impact on downstream genes. Color, shade, and shape indicate key driver, module membership, and PBMC asthma genes as summarized in the legend. Some genes downstream of key drivers are additionally highlighted given their recognized roles in immune-related functions. **B** Functional biological context for the interleukin production module. Genes highlighted within blue boxes are key drivers of the module. (1) *CTSS*, Cathepsin S, is a lysosomal cysteine proteinase that cleaves off the invariant chain on MHC class II molecules in the endolysosomal compartments for later antigen-MHC II formation [60]. *ATP6AP2* encodes a protein involved in lysosomal proton-transporting V-type ATPase [55]. (2) *TNFSF13* encodes a member of the tumor necrosis factor ligand superfamily important for B cell development [61]. (3) *RAB3D* encodes a member of the RAS oncogene family that regulates secretory granule maturation [56]. (4) *PSAP* encodes prosaposin, which yields Saposin B when cleaved [50]. Saposin B works with other enzymes to break down sphingolipids [52]. (5) *CAPZA2*, capping actin protein of muscle Z-line subunit alpha 2, is an F-actin capping protein that caps the barbed end of actin filaments [57]. (6) *LCP1* encodes L-plastins (LPL) that bind F-actin. LPL also mediates sensitization of eosinophils [51]

The PBMC key drivers for the NK cell module are shown in Fig. 3A and for the interleukin module in Fig. 4A. As key drivers predicted to exert the greatest causal impact on downstream genes in each module, these key drivers appear at the top of each Eiffel tower plot, with causality flowing from top to bottom. In addition to the key drivers, we label a few downstream genes of interest for each module. Biological relationships between the key drivers for each module, based on the probabilistic causal network and prior experimental work, are shown in Figs. 3B and 4B [46–61].

### Local airway processes in asthma: nasal asthma genes, nasal asthma modules, and nasal key drivers

To characterize local airway processes in asthma, we next performed the same analytic flow with the nasal transcriptome data [44] that had been generated in parallel with the PBMC data (Fig. 1). The same discovery and test set assignments were used for the available nasal transcriptome data. Nasally expressed genes found to be associated with asthma by differential expression analysis in the discovery set that were also significantly associated with asthma in the independent test set were deemed “nasal asthma genes” (Additional file 1: Table S10).

To explore how immune cells in the nasal samples might influence the nasal asthma genes identified, we performed cellular deconvolution [29] and found small cell fractions of myeloid, mast cells, and plasma cells in the nasal samples that did not differ between subjects with and without asthma (Additional file 1: Table S11). Adding these cell fractions as additional model covariates did not significantly change the nasal genes identified; nasal asthma genes from this sensitivity analysis strongly overlapped with those found from the original model (enrichment score 12.7, Fisher’s exact *p* value = 6.3e − 306).

WGCNA using the nasal transcriptome discovery set identified 21 nasal modules, of which 8 were significantly enriched with nasal asthma genes (FDR ≤ 0.05). Of these 8 modules, 2 had module eigenvalues significantly associated with asthma at FDR ≤ 0.05: the module for tricarboxylic acid (TCA) cycle and the module for metabolic pathways (Additional file 2: Fig. S1). The TCA module eigenvalue was associated with asthma with greatest effect size and lowest FDR (Additional file 2: Fig. S1) and was therefore considered the top ranked “nasal asthma module.” The metabolic pathways module, whose eigenvalue was also nominally associated with asthma but with lower effect size and higher FDR, was considered a broad module representing cellular maintenance.

Construction of a probabilistic causal network with the nasal transcriptome discovery set and key driver analysis for the TCA nasal asthma module identified nasal key drivers. An independent probabilistic causal network was then built with the nasal transcriptome test set with key driver analysis also performed. Key drivers identified for the TCA nasal asthma module in the discovery set (Additional file 1: Table S12) that were also validated in the test set were considered “nasal key drivers.” These nasal key drivers included *G3BP1* and *INADL*, two members of the TCA module that were also nasal asthma genes themselves (Additional file 1: Table S10).

### Relationship between systemic and local processes in asthma: nasal key drivers causally mediate associations between PBMC key drivers and asthma

**Fig. 5:**
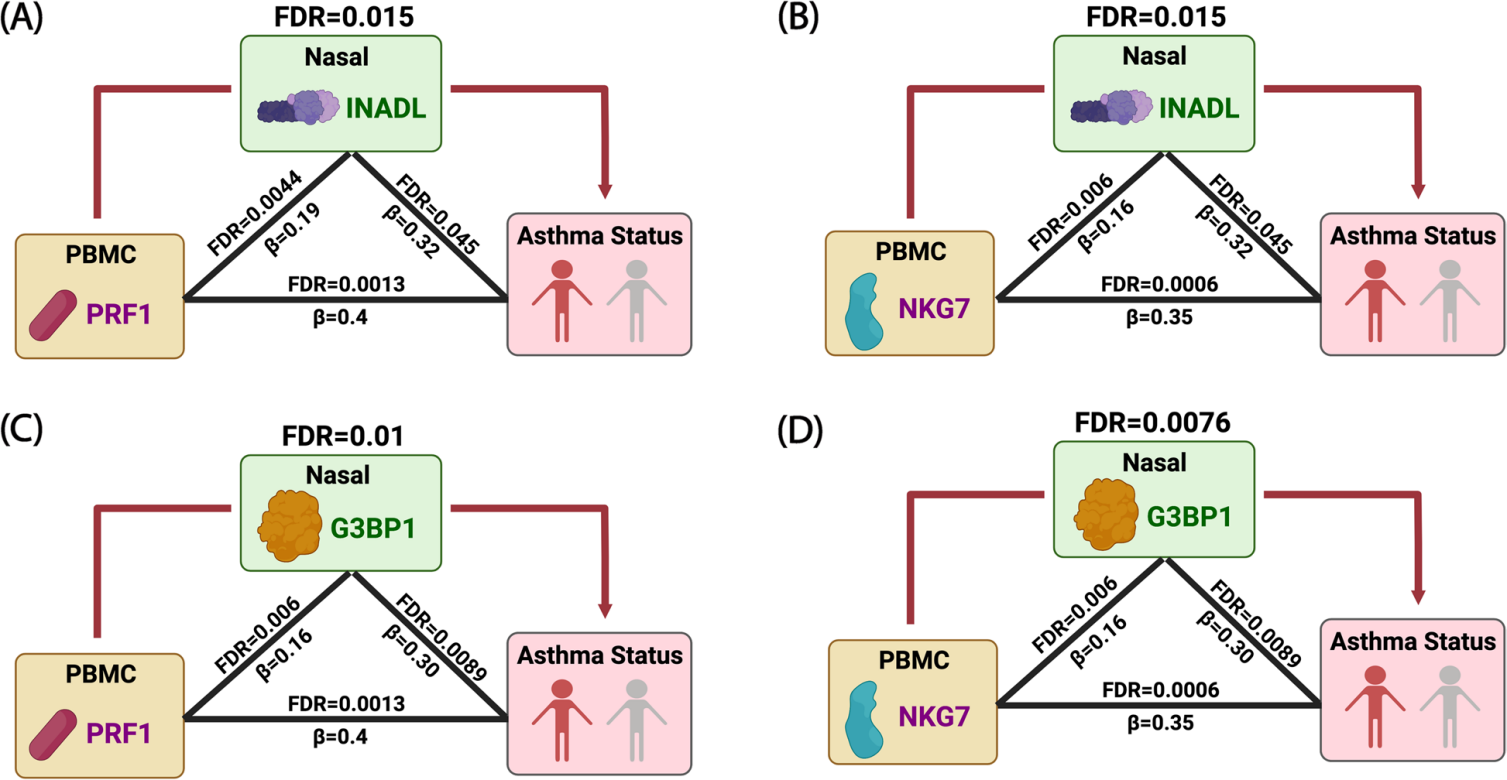
Associations between PBMC key drivers and asthma are causally mediated by nasal key drivers. PBMC key drivers and nasal key drivers associated with asthma were examined. Triangle edges summarize pairwise associations. Red arrows indicate significant causal mediation by nasal key drivers (FDR $\le$ 0.05)

Our next goal was to investigate the relationship between systemic and local processes in asthma. Specifically, we sought to examine causal mediation between PBMC key drivers associated with asthma (*PRF1* and *NKG7* in the NK cell module; *CAPZA2*, *ATP6AP2*, *CTSS*, and *RAB3D* from the interleukin module) and nasal key drivers associated with asthma (*G3BP1* and *INADL* from the nasal TCA module). These causal mediation models revealed that the nasal key drivers of asthma significantly mediate the association between PBMC key drivers in the NK cell module (*PRF1* and *NKG7*) and asthma (FDR = 0.0076 to 0.01) (Fig. 5). No causal mediation was observed in the interleukin module, and there was no statistically significant finding for the converse of PBMC key drivers mediating associations between nasal key drivers and asthma. Permutation testing [62] with 1,000,000 iterations confirmed our finding of nasal key drivers significantly mediating associations between PBMC key drivers and asthma in the NK cell module and lack of significance for mediation by PBMC key drivers.

## Discussion

In this integrated study of systemic and local processes in asthma, we generated and leveraged parallel PBMC and nasal transcriptome profiles [44] from 341 individuals in a well-characterized asthma cohort to identify systemic and local key drivers of asthma, as well as causal mediation relationships underlying systemic-local cross-talk in this complex disease. By capturing both systemic and local transcriptomics and employing innovative network analyses, this study moves beyond prior transcriptomic studies of asthma by (1) finding evidence for both systemic and local transcriptional signatures of asthma in the same cohort (Additional file 1: Table S5, Additional file 1: Table S10); (2) identifying key drivers of these asthma signatures in the PBMC and airway compartments (Figs. 3 and 4, Additional file 1: Table S8, Table S9, Table S12); and (3) characterizing causal relationships between the detected systemic and local key drivers (Fig. 5). The discovery and test set approach that we employed, where each discovery step required validation in a test set of independent participants, brings rigor to the findings reported.

Through differential gene expression, WGCNA, and module enrichment analyses, we found that NK cell-mediated cytotoxicity and interleukin production were the biological processes for the top PBMC modules enriched for asthma-associated PBMC genes, while TCA cycle was the top nasal module enriched for asthma-associated nasal genes. These findings from unbiased whole transcriptome analyses of the PBMC and nasal compartments highlight mechanistic pathways that have to date been overshadowed by focus on commonly targeted pathways (e.g. type 2 inflammation) [63]. The importance of these biological processes is supported by prior findings. For example, increased NK cell cytotoxicity activity is present in the peripheral blood of asthmatics vs. controls, and children with acute exacerbated asthma have higher numbers of circulating NK cells compared to those with stable asthma [64]. NK cells activate in response to well-known allergy triggers including allergens, RSV, and other respiratory viruses [64]. Furthermore, compared to controls, patients with asthma have increased IL-4-producing NK cells in their blood [64]. Our finding of interleukin production as a top PBMC asthma module is not surprising, given the broad representation of this module and the pervasive roles that interleukins play in orchestrating inflammatory processes in asthma, including IL-4, IL-5, and IL-13 in type 2 inflammation [65, 66]. Indeed, the pivotal role of circulating interleukins has been the basis for the explosion of biologic medications as effective systemic treatments for asthma [67]. Moreover, we found significant enrichment of Th2 cell differentiation genes in the interleukin PBMC asthma module. In contrast, finding that the TCA module was the top nasal asthma module was less expected, although evidence from the cellular and molecular biology field supports that members of the TCA cycle act mechanistically to shape smooth muscle airway bronchoconstriction, hyperresponsiveness, and airway remodeling in asthma [68, 69].

While illuminating to identify genes and modules associated with asthma in both the PBMC and nasal compartments, we thought it would be important to move beyond association toward new insight on key drivers of systemic and local processes in asthma. The identification of key drivers prioritizes gene signature lists by highlighting the transcripts at root levels of causality for more efficient therapeutic targeting and mechanistic parsing. Here, we mapped and leveraged expression quantitative trait loci as priors to build probabilistic causal networks with directional connectivity, which then enabled us to identify key drivers of the PBMC and nasal asthma modules (Figs. 3 and 4, Additional file 1: Table S8, Table S9, Table S12). These key drivers are statistically predicted to drive the regulatory state of each asthma module. For the NK cell PBMC asthma module, we identified perforin (*PRF1*) and NK cell granule protein (*NKG7*) as key drivers that were each also associated with asthma (Fig. 3A). Increased expression of *PRF1* in peripheral blood lymphocytes of both allergic and intrinsic asthmatics has been previously reported, [70] and *NKG7* was found in a study of publicly available gene expression data to be a marker of severe asthma [71]. Both PRF1 and NKG7 demonstrated prominent upstream roles in NK cell-mediated cytotoxicity when causal relationships derived from the constructed network and evidence from the literature were combined to provide functional biologic context (Fig. 3B). Given the broader scope of the interleukin production PBMC asthma module, more key drivers were identified for this module (Fig. 4A), which were predicted by the causal network and prior literature to regulate a wide variety of cellular activities including those involved in prevalent type 2 asthma, such as allergen presentation and eosinophil chemotaxis (Fig. 4B). For the nasal asthma module, G3BP stress granule assembly factor 1 (*G3BP1*), which promotes innate immune transcriptional responses via NF-kB and c-Jun N-terminal kinase pathways, [72] and the protein-coding gene InaD-like protein (*INADL*) involved in epithelial migration [73] were both identified as nasal key drivers that were also associated with asthma.

In contrast to prior investigations of transcriptomic associations with asthma and asthma-related phenotypes in blood only [17–19], upper airway samples only [6–11], and both where only airway but no systemic associations were found [20, 21], we found evidence for both systemic and airway transcriptomic associations with asthma in the same cohort. Our sample size of 341 participants was relatively large for a dual transcriptome study [12, 20, 21], augmenting our power for detection and also enabling the network and discovery/test set approaches we used to ensure rigor and advance insight beyond associations. Our paired findings from the systems and local domains provided us with a unique opportunity to begin to address the fundamental question of the degree to which asthma is a local process with systemic findings or a systemic process with local findings. While this is a somewhat existential question without an absolute answer, our causal mediation analyses of the relationships between the identified PBMC asthma key drivers, nasal asthma key drivers, and asthma status in this population begin to offer some insight. Here, we found that associations between PBMC key drivers and asthma were causally mediated by nasal key drivers, but not the converse (Fig. 5). Specifically, for the PBMC module key drivers *PRF1* and *NKG7*, their association with asthma was causally mediated by the nasal asthma key drivers *G3BP1* and *INADL*. There was no evidence for the converse of PBMC key drivers causally mediating associations between nasal key drivers and asthma. This suggests that asthma is a systemic process causally mediated by airway key drivers. Given circulating immune cells can be recruited to the airway during inflammation [13, 16], our findings speak to the cross-talk that bridges systemic and local inflammation and immunity. While we found significant causal mediation relationships for PBMC key drivers of the NK cell module (*PRF1* and *NKG7*), there were no detectable causal mediation effects for PBMC key drivers of the interleukin module, which may have been due to the broader, less specific scope of that module.

We recognize the limitations to our study. While our investigation of parallel PBMC and nasal transcriptome profiles in asthma stands apart in successfully finding both systemic and airway gene signatures, modules, and key drivers of asthma as well as causal relationships between them, our study did not endotype asthma. It is possible that different findings would be detected if analyses were limited to particular endotypes of asthma, which was outside the scope of this study but a future direction we will consider. Additionally, while we employed a discovery and test set approach where each was analyzed independently at every step to ensure rigor in our reported findings, the discovery and test sets were sampled from the same population. It is possible that findings would be distinct for asthma populations in other parts of the world. Finally, the key drivers were identified via mathematical and statistical models, and we acknowledge that further work is needed to replicate and experimentally validate these findings to deepen our understanding of their role in asthma.

## Conclusions

In this network study of systemic and local processes in asthma, we generated and examined dual PBMC and nasal transcriptome profiles from 341 individuals to identify systemic and local key drivers of asthma, as well as causal mediation relationships between them. In addition to identifying both systemic and local transcriptional signatures of asthma, we found that associations between systemic key drivers of asthma and asthma are causally mediated by nasal key drivers of asthma. Our study moves beyond anatomically isolated transcriptome-wide association analyses to elucidate causal relationships between systemic and airway key drivers of asthma.

### Supplementary Information

**Additional file 1:** **Table S1.** Characteristics of the participants who provided nasal samples. **Table S2.** NK cell-mediated cytotoxicity PBMC module eQTLs. **Table S3.** Interleukin production PBMC module eQTLs. **Table S4.** Nasal TCA cycle module eQTLs. **Table S5.** PBMC asthma genes. **Table S6.** PBMC NK cell-mediated cytotoxicity module genes. **Table S7.** PBMC interleukin production module genes. **Table S8.** Key driver analysis results for the PBMC NK cell-mediated cytotoxicity module. **Table S9.** Key driver analysis results for the PBMC interleukin production module. **Table S10.** Nasal asthma genes. **Table S11.** Nasal Myeloid, Mast cell and Plasma cell fractions. **Table S12.** Key driver analysis results for the nasal TCA cycle module.

**Additional file 2:** **Fig. S1.** Associations between nasal module eigenvalues and asthma.

## Data Availability

Data for this study, including PBMC and nasal RNAseq readcount tables, are available at https://doi.org/10.7303/syn51061925 [44] on Synapse.
