# Supplementary material for: Integrated study of systemic and local airway transcriptomes in asthma reveals causal mediation of systemic effects by airway key drivers
Source: Genome Med. 2023 Sep 20;15:71. doi: 10.1186/s13073-023-01222-2 (PMC10512627; doi:10.1186/s13073-023-01222-2)
Supplement: Supplementary file 2 — Additional file 2: Fig. S1. Associations between nasal module eigenvalues and asthma. [file 13073_2023_1222_MOESM2_ESM.docx]

**Fig. S1.** Associations between nasal module eigenvalues and asthma
